# An Inhibitory Sex Pheromone Tastes Bitter for *Drosophila* Males

**DOI:** 10.1371/journal.pone.0000661

**Published:** 2007-08-15

**Authors:** Fabien Lacaille, Makoto Hiroi, Robert Twele, Tsuyoshi Inoshita, Daisuke Umemoto, Gérard Manière, Frédéric Marion-Poll, Mamiko Ozaki, Wittko Francke, Matthew Cobb, Claude Everaerts, Teiichi Tanimura, Jean-François Ferveur

**Affiliations:** 1 Université de Bourgogne, CNRS-UMR5548, Dijon, France; 2 INRA-UMR1272,Versailles, France; 3 Organic Chemistry, Hamburg University, Hamburg, Germany; 4 Department of Biology, Graduate School of Sciences, Kyushu University, Fukuoka, Japan; 5 Department of Applied Biology, Faculty of Textile Science, Kyoto Institute of Technology, Kyoto, Japan; 6 Faculty of Life Sciences, University of Manchester, Manchester, United Kingdom; Northwestern University, United States of America

## Abstract

Sexual behavior requires animals to distinguish between the sexes and to respond appropriately to each of them. In *Drosophila melanogaster*, as in many insects, cuticular hydrocarbons are thought to be involved in sex recognition and in mating behavior, but there is no direct neuronal evidence of their pheromonal effect. Using behavioral and electrophysiological measures of responses to natural and synthetic compounds, we show that *Z*-7-tricosene, a *Drosophila* male cuticular hydrocarbon, acts as a sex pheromone and inhibits male-male courtship. These data provide the first direct demonstration that an insect cuticular hydrocarbon is detected as a sex pheromone. Intriguingly, we show that a particular type of gustatory neurons of the labial palps respond both to *Z*-7-tricosene and to bitter stimuli. Cross-adaptation between *Z*-7-tricosene and bitter stimuli further indicates that these two very different substances are processed by the same neural pathways. Furthermore, the two substances induced similar behavioral responses both in courtship and feeding tests. We conclude that the inhibitory pheromone tastes bitter to the fly.

## Introduction

Sexual behavior requires animals to distinguish between the sexes and respond appropriately to each of them [1]. The genetic potential of *Drosophila melanogaster* has made it a focus of study to investigate the role of genes that affect male courtship behavior and sexual orientation [2]. Most studies have centred on the stereotyped male courtship, rather than on the range of sensory cues that are integrated to produce changes in the male's behavior [3]. In *D.melanogaster*, as in many insect species, chemical signals likely play a key role in sex recognition, and in the initiation and progress of courtship [4], [5]: males are thought to be excited by a range of chemical substances produced by the females, including long-chain hydrocarbons, and to be inhibited by hydrocarbons on the male cuticle, especially by *Z*-7-tricosene (7-T) [6].

These substances, which have low or very low volatility [7], [8] are thought to be detected by gustatory receptors (Gr) on the male's legs and proboscis [4], [9], but there is no direct neuronal evidence of their pheromonal role. The strongest evidence thus far obtained comes from the manipulation of male-specific neurons expressing the GR68A gustatory receptor protein, found in gustatory sensilla on fore-tarsi, which apparently altered the duration of male courtship of females [10]. In support of this finding, a putative pheromone-binding protein expressed specifically in these gustatory sensilla has been shown to be required for male discrimination of sex objects [11]–[13]. Strikingly, the pheromone(s) causing these effects has not been identified, nor has the nature of the responses shown by these sensilla been determined.

Cuticular hydrocarbons are lipophilic and difficult to manipulate using the classical electrophysiological “tip-recording” method, which uses a single electrode filled with hydrophilic solution eventually mixed with a detergent [14], [15]. We designed a device consisting of two electrodes, with which we could separately stimulate taste sensilla and record their physiological activity in response to synthetic 7-T (tungsten electrod method). We also investigated the pheromonal effect of pure 7-T on male courtship intensity. We found that (i) the same taste neurons respond to both 7-T and to bitter substances and (ii) these two types of molecules similarly inhibit male behavior in a dose-dependent manner.

## Results

### 7-T induces dose-dependent inhibition of male homosexual courtship

**Figure 1:**
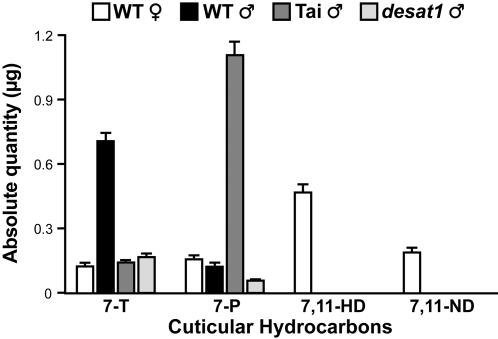
Levels of the principal cuticular hydrocarbons in flies of various genotypes. Data shown represent the mean (±sem) for absolute amount of the principal hydrocarbons detected on the cuticle of 5-day-old flies. 7-T = *Z*-7-tricosene; 7-P = *Z*-7-pentacosene; 7-11 HD = *Z-Z*-7,11-heptacosadiene; 7-11 ND = *Z-Z*-7,11-nonacosadiene. The genotypes shown correspond to the target flies tested either with a WT male or with a *Gr66a-Gal4*/WT male (see Fig. 2A). WT = Canton-S strain.

**Figure 2:**
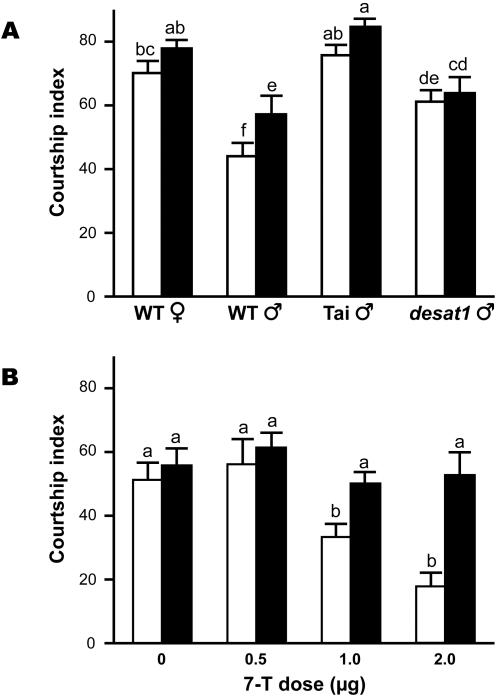
* Z-*7-tricosene (7-T) inhibits male courtship behavior. Histograms represent the mean courtship index (CI±sem) in tester males from the wild-type Canton-S strain (WT; empty bars) and the *Gr66a-Gal4/*WT genotype (filled bars) during 10 min under red light. Tests always involved a pair of 5-day-old flies: an intact tester male and a decapitated target fly. (A) CIs toward decapitated target flies of four genotypes: WT females and males, Tai and *desat1* males. The relatively high homosexual CI shown by WT males toward sibling males can be imputed to the elimination of inhibitory visual and acoustic signals that are emitted by intact males under white light [16], [17]. The principal cuticular hydrocarbons of these flies are shown on Fig. 1. (B) All tester males courted target *desat1* mutant males covered with different doses of synthetic 7-T. The doses shown—0.5 µg, 1.5 µg and 2 µg—respectively correspond to 4, 12 and 16 mM 7-T. “0” corresponds to the solvent (pentane) used to dissolve 7-T. A similar amount of solvent was used for all tests (0.8 µl). All data were Arcsin transformed and tested using two-factors ANOVA and PLSD Fisher test. For each data set, differences were tested between subjects, between objects and for the interaction between factors: for A, *p* = 0.004; 0.001; 0.863, respectively; for B, *p* = 0.002; 0.001; 0.098, respectively. Significant difference (*p*<0.05) for each set of data are indicated by letters above each bar (same letters = no difference); n = 25–30.

To assess the effect of sex pheromones on male courtship behavior, we measured the courtship index (CI) that wild-type Canton-S (WT) males directed towards immobilized target flies with various cuticular hydrocarbon profiles (Fig. 1). All tests were carried out under dim red light and with decapitated targets so as to enhance the behavioral effect of pheromones (see Materials and Methods) [16], [17]. Under these experimental conditions, WT males produced relatively high levels of homosexual courtship (CI = 44.1±4.1; Fig. 2A; empty bars) to sibling target WT males which produce high levels of 7-T (Fig. 1) However, as expected, these tester males showed significantly higher CIs toward WT females, and also to Tai and *desat1* males. These three types of fly all have low levels of 7-T. However, in other respects they have different cuticular hydrocarbon profiles—Tai males (from West Africa) are rich in *Z*-7-pentacosene (7-P, a stimulatory pheromone for *D. melanogaster* males [8]), whereas males from the *desat1* mutant line have very low levels of 7-P [18]. WT females have also very low levels of 7-P but produce high levels of 7,11 heptacosadiene (7,11-HD) and 7,11 nonacosadiene (7,11-ND) which tend to enhance male courtship stimulation [6]–[8].

These data confirm previous suggestions [6], [8], [19] that 7-T tends to inhibit homosexual courtship by WT males. To demonstrate this, we synthesized 7-T [20] and placed various doses of this substance on the cuticle of a *desat1* mutant male which show almost no 7-T (Fig. 2B). The results further supported our hypothesis: tester WT males showed a negative correlation between their courtship of 7-T covered *desat1* males, and the dose of synthetic 7-T placed on the target male. 0.5 µg 7-T did not affect the CI whereas 1 µg 7-T—which roughly corresponds to one WT male-equivalent—induced a CI that was similar to that induced by WT target males, and 2 µg 7-T induced a strong inhibition of WT male courtship. The solvent (pentane) used to dissolve 7-T had no significant effect on male courtship. These data demonstrate that 7-T induced a dose-dependent inhibition of the homosexual courtship shown by WT tester males.

### 7-T elicits a dose-dependent physiological response in proboscis gustatory neurons

**Figure 3:**
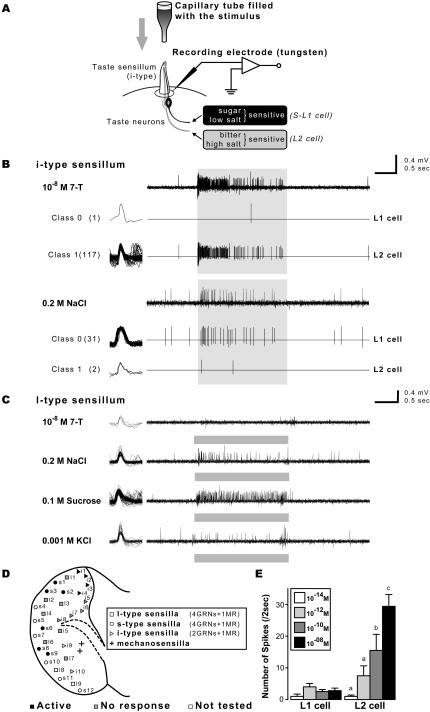
7-T elicits a dose-dependent electrophysiological response in a subset of labial palp gustatory neurons. (A) Schematic illustration of the “tungsten electrod method” which allows simultaneous but separate stimulation and recording of gustatory and pheromonal stimuli. The stimulus was contained in a glass microelectrode capping the tip of the sensillum; caffeine and 7-T were dissolved in mineral oil, sucrose and salts were dissolved in water. Recordings were obtained from three types of labellar sensilla (l-, i- and s-type) determined according to both their location and shape [21]–[23] in wild-type Canton-S males. So far, we were unable to use this method to record from sensilla on the fore-tarsus, because of the relative hardness of the leg cuticle and the slender form of this appendage. (B) In i-type sensilla, one cell responded to 10^−8^M 7-T, whereas the second cell responded to 0.2 M NaCl. The shaded bar represents the duration of the stimulation (2 sec). Elicited spikes were separated according to spike height (amplitude of “class 0” and “class 1” were 0.3–0.5 mV and 0.1–0.2 mV, respectively); see left side of panel; n = 20 flies. (C) Conversely, l-type sensilla responded normally to NaCl, sucrose, KCl but not to 10^−8^M 7-T. Recording were made in similar conditions as described for i-type sensilla; n = 32 flies. (D) 7-T responsive sensilla were mapped on the labial palp with tungsten electrode recordings. Both i- and s-types-but no l-type—sensilla responded to stimulation with 7-T (a sensillum was considered as responsive if it showed at least one response to 7-T among 2-5 trials). Anterior is up, dorsal is right. GRN = gustatory receptor neuron; MR = mechanoreceptor neuron. (E) In a s-type sensillum (S2), the L2 cell showed a dose-dependent increased activity to 7-T (between 10^−12^ and 10^−8^M) while the L1 cells were not activated. Vertical axis: total number of spikes during 2 sec stimulation. Significantly increased activity of the L2 cell is indicated (a–b: *p*<0.05, a–c: *p*<0.01; n = 4–10).

To investigate the role of gustatory neurons in detecting male inhibitory pheromones, we measured the electrophysiological activity of these neurons in response to pure synthetic 7-T. Our tungsten electrode method allowed us to separately stimulate and record the electrophysiological activity of a sensillum on the labial palp. The stimulating electrode was filled with a lipophilic buffer (paraffin oil) containing 7-T, and capped the tip of the taste sensillum, while the tungsten recording electrode was inserted at the base of the sensillum (Fig. 3A).

We recorded from the three types of sensilla present on the labial palps: i-type (which have two gustatory receptor neurons), s- and l-type taste sensilla (which have four gustatory receptor neurons [21]–[23]), and mapped the sensilla according to their physiological responses (Fig. 3B–D). Six out of six s- and four out of eight i-type sensilla so far examined consistently responded to 7-T at the 10^−8^M concentration (Fig. 3B, D). This provides the first direct evidence of a neuronal response to a cuticular hydrocarbon sex pheromone in an insect. In i-type sensilla, only one of the two gustatory neurons responded to 7-T. The active cell was the L2 cell, which produced small spikes (Fig. 3B). In contrast, the second gustatory neuron (the L1-S or L1 cell), housed in the same i-type sensilla, did not respond to 7-T but responded normally to salt (larger spikes; Fig. 3B) and sucrose (data not shown). The firing frequency of the L2 cell, measured in a s-type sensilla, increased with 7-T concentration (between 10^−12^ and 10^−8^ M; Fig. 3E). Conversely, l-type sensilla on the labial palp responded to NaCl, KCl and sucrose, but not to 10^−8^M 7-T (Fig. 3C).

### A single class of gustatory neurons responds to both 7-T and caffeine

**Figure 4:**
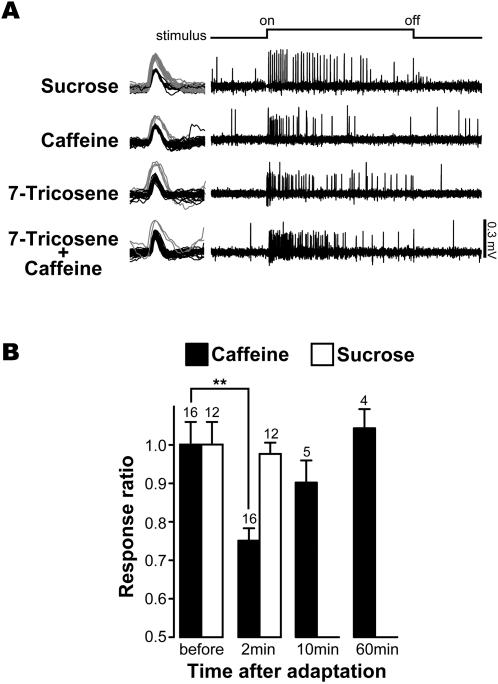
Caffeine and 7-T are detected by the same gustatory sensory neuron in i-type sensilla. (A) Above: 50 mM sucrose stimulated the L1 cell (on the left, spikes shown in gray = 0.2–0.3 mV). Below: in the same sensillum, caffeine excited the L2 cell, while 10^−8^M 7-T elicited a single class of spikes with an amplitude equivalent to those from the L2 cell (black spikes shown on the left = 0.1–0.2 mV). Bottom: a mixture of caffeine and 7-T (with similar concentrations as above) elicits high levels of activity in a single class of spikes. This indicates that 7-T activates the L2 cell but not the L1 cell. Recordings are shown for 4 sec and the stimulus application lasted for 2 sec. n = 10 (obtained with 5 male flies). (B) Caffeine and 7-T show cross-adaptation. Neuronal responses to caffeine were reduced following pre-stimulation with 7-T, supporting the hypothesis that 7-T and caffeine are detected by the same taste neuron. This effect was highly significant two minutes after pre-stimulation with 7-T (**: *p* = 0.0016; two tailed paired t-test). In contrast, the response to sucrose remained unaffected by pre-stimulation with 7-T. The response ratio was calculated from the number of spikes induced by a given substance, before and after prestimulation. The filled bar represents the response of the L2 cell to caffeine; the empty bar represents the response of the L1 cell to sucrose. The numbers represent the number of stimulated sensilla (each recording was made with a different fly).

The L2 cell in i-type sensilla is known to respond to bitter molecules [22], [23]. To investigate whether the same neuron processes both 7-T and bitter substances, we carried out a series of experiments. When stimulated with a mixture of 7-T and caffeine, the L2 cell elicited an increased number of spikes with the same amplitude than when stimulated with either substance alone (Fig. 4A). 7-T and bitter substances also show cross-adaptation: pre-stimulation with 7-T significantly reduced the response of L2 cells to caffeine but not to sucrose (Fig. 4B). Taken together, these additive and cross-adaptative effects strongly indicate that 7-T and bitter stimuli are processed by the same taste neuron, the L2 cell, in the responsive i-type sensilla of the labial palps.

### Bitter molecules inhibit male courtship and 7-T inhibits feeding responses

**Figure 5:**
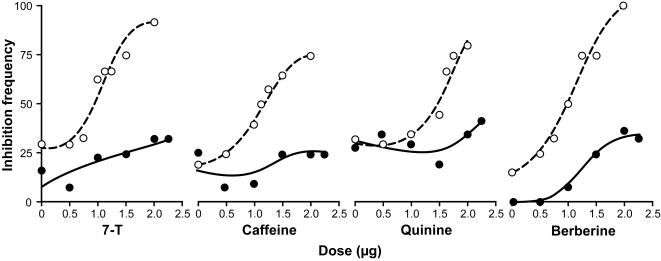
7-T and of bitter molecules dose-dependently inhibit male courtship. The intensity of courtship (CI) was measured in tester WT males (empty circles) and in *Gr66a-Gal4*/WT males (filled circles) paired with decapitated *desat1* target males covered either with pure 7-T or with one of the three bitter molecules—caffeine, quinine or berberine—as indicated below the curves. Each chemical to be tested was diluted in the appropriate solvent at a concentration adjusted to standardize the volume of solvent deposited on each object fly (see Material and methods). The solvents used here had no effect on male CI. The frequency of inhibition corresponds to the number of males with CI<44.1 (observed in the control situation between two WT males). For example, 50% inhibition indicates that 50% of the tester males showed a CI<44.1. n = 20–30.

To confirm this apparent dual sensory processing at the neuronal level, we observed the reciprocal cross-effects (*i*) of bitter stimuli on sexual behavior and (*ii*) of 7-T on feeding-related behavior. Painting *desat1* males with any of three bitter substances–caffeine, quinine or berberine–strongly inhibited male courtship of these painted flies by WT control males (Fig. 5; empty dots). Tester males were sensitive to these bitter stimuli just as they were to synthetic 7-T: the three bitter substances induced dose-dependent effects very similar to those induced by 7-T (Fig. 5). Berberine induced a more potent inhibition than the two other bitter substances: 1 µg and 2 µg of the former molecule respectively inhibited 50% and 100% males; similar doses of the latter molecules respectively inhibited 35 and 80% males. The three solvents used to dissolve each bitter molecule did not affect homosexual CI (see Materials and Methods). These results clearly show that bitter substances induce dose-dependent inhibition of male homosexual courtship.

**Figure 6:**
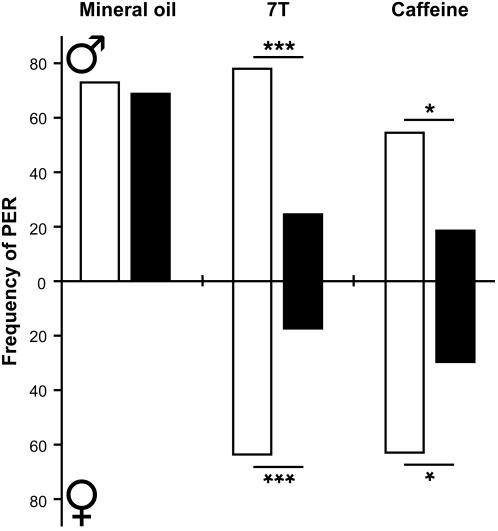
7-T and caffeine both inhibit the proboscis extension reflex (PER) of male and female flies. Data indicate the PER frequency in male and female flies either unilaterally stimulated with 0.1M sucrose (empty bars), or bilaterally stimulated with 0.1M sucrose on one side and a test solution on the other side (filled bars). The test solution either consisted of 10^−8^M 7-T, or 0.1M caffeine (both dissolved in mineral oil), or mineral oil (tested only in males). Significant differences in PER between the two stimulation conditions were estimated for each treatment and sex by Chi2 with Yates' correction (*** : *p*<0.001 ; * : *p*<0.05). Each data set was obtained from 6–7 series of experiments with 70–90 flies except for caffeine (n = 22–30).

To assess whether the sensory processing of 7-T affected feeding behavior, we performed the Proboscis Extension Reflex (PER; [24]). When sensilla on the tarsi were unilaterally stimulated with 0.1M sucrose, a positive PER was shown by a majority of male and female flies (Fig. 6). When flies were bilaterally stimulated, on one side with 0.1M sucrose and on the contralateral side with 10^−8^M 7-T, PER was highly reduced in both sexes (*p*<0.0001). PER was also significantly reduced (p<0.05) in both sexes by a contralateral application of 0.1M caffeine, whereas the solvent (mineral oil) used to dissolve 7-T had no effect. These data are supported by a previous PER experiment carried out with berberine [22]. Together, they indicate that both 7-T and bitter compounds similarly affect the appetitive behavior of male flies.

### Manipulation of a subset of taste neurons affects courtship inhibition by 7-T and by bitter molecules

Finally, we measured courtship behavior in males with genetically altered taste neurons. To target neurons potentially involved in the perception of bitter substances and of 7-T, we used the *Gr66a-Gal4* transgene which contains the promoter of the gustatory receptor *Gr66a* gene fused with the yeast *Gal4* sequence [25]. This choice was based on two observations: firstly, both the GR66A-receptor protein and *Gr66a-Gal4*-expressing neurons are involved in the detection of caffeine [25], [26]. Second, using *UAS*-*GFP*, the expression of the *Gr66a-Gal4* transgene was visualized and found in approximately 22 i- and s-type taste sensilla symmetrically arranged on each labial plate (these were the same sensilla in which we previously observed a dual response to 7-T and caffeine) and in 7 to 8 taste sensilla on each front leg. In the labial palps, a single neuron in each sensillum expressed GFP under the control of *Gr66a-Gal4* (data not shown).

To assess the effect of *Gr66a*-*Gal4* expressing neurons on male courtship and taste perception, we measured the courtship index (CI) that males with targeted *Gr66a-Gal4* expressing neurons directed towards immobilized target WT males. When we deleted *Gr66a*-*Gal4* expressing cells using the pro-apoptotic transgene *reaper* (*UAS*-*rpr*), *Gr66a*-*Gal4*; *UAS*-*rpr* males showed a significantly enhanced homosexual CI to WT target males (CI = 61.6±5.8; two sided t test, *p* = 0.002) if compared to the CI shown by control WT males toward similar target males. The amplitude of this effect was similar to that shown by *Gr66a-Gal4*/WT males carrying the *Gr66a-Gal4* transgene alone (CI = 56.7±5; two sided t test, *p* = 0.096), indicating that the GAL4 protein directly or indirectly affects the targeted taste cells and changes pheromonal perception. Strikingly, the effect of the transgene alone was specific to WT target males: the CIs of *Gr66a-Gal4*/WT tester males toward WT females and toward Tai and *desat1* males were not significantly different to those shown by WT tester males (Fig. 2; filled bars), reinforcing the suggestion that *Gr66a-Gal4*-expressing neurons are specifically involved in detecting inhibitory chemical stimuli.

We then measured the inhibitory effect induced by pure 7-T on *Gr66a-Gal4*/WT males using *desat1* target males painted with various amounts of 7-T. The results further supported our hypothesis: contrary to the strong dose-dependent effect induced in WT males, 7-T had a much weaker—if any—inhibitory effect on *Gr66a-Gal4*/WT flies (Fig. 2B; filled bars). For example, 2 µg 7-T inhibited most WT males whereas a similar dose inhibited only 25% of *Gr66a*-*Gal4*/WT males (Fig. 5; filled dots). As with the effects of 7-T, the three bitter molecules had less–if any–effect on *Gr66a-*Gal4/WT males compared to WT males. Taken together, these data suggest that *Gr66a-Gal4* expressing neurons are required to detect the aversive effect induced by 7-T and by bitter stimuli on male courtship behavior.

## Discussion

By combining behavioral, chemical, electrophysiological and genetic approaches we have provided the first direct neuronal evidence of cuticular hydrocarbon sex pheromone processing in an insect. We have also shown that an inhibitory sex pheromone and repulsive gustatory stimuli are processed by the same neurons (The L2 cells corresponding to some *Gr66a-Gal4* expressing neurons) and induced similar behavioral responses. For the male fly, there is apparently no difference between the sensation induced by bitter stimuli and that induced by the inhibitory pheromone 7-T.

Given that the two types of substances have little structural similarity–7-T is a straight chain hydrocarbon whereas berberine, caffeine, and quinine are oxygenated, oligocyclic alkaloids–it is unlikely that they are detected by the same receptor molecule. Since multiple types of GR molecules are probably co-expressed in *Gr66a-Gal4* expressing labellar taste neurons [25], [27], and the GR66A receptor molecule is involved in the detection of caffeine [26], this suggests that 7-T and perhaps other bitter substances are detected by other, unknown receptor molecule(s). In this case, cross-adaptation would presumably take place through common activation of second-messenger systems or of calcium trafficking [28].

It is not yet clear how GAL4 alters the activity of *Gr66a*-*Gal4-*expressing neurons. Recent results have shown that GAL4 can have a toxic effect on neurons [29]. This could also explain previous findings in which male flies carrying a single copy of a Gal4 transgene targeting taste sensilla showed altered pheromonal responses [12].

Our findings provide experimental support for Darwin's hypothesis that sexual selection operates on pre-existing structures and behaviors, co-opting them into new functions [1]. In the present case, we propose that pre-existing neuronal networks responsible for detecting and responding to bitter substances became able to detect stimuli produced by other males, with the result that these stimuli inhibited male courtship by activating aversive behaviors that were previously solely induced by bitter stimuli. A similar—likely convergent—process may have taken place in vertebrates, when some semiochemicals produced by male mice are detected by specialized receptor molecules in the main olfactory bulb, not in the accessory olfactory system, which had previously been considered to be the sole site of pheromone processing [30], [31]. We have shown that identical peripheral neurons are involved in detecting inhibitory pheromones and aversive gustatory stimuli. The next challenge will be to understand how these signals are represented in the fly brain.

## Materials and Methods

### Fly Husbandry, Genetics and Behavior

All *D. melanogaster* strains were raised on yeast/cornmeal/agar medium and kept at 24±0.5°C and 65±5% humidity on a 12:12 h light/dark cycle. All crosses involving the *Gr66a-Gal4* transgene were carried out between females homozygous for the *Gr66a-Gal4* transgene and either WT (Canton-S) males or homozygous males carrying the *UAS-rpr* transgene. Courtship tests were performed on the F1 male progeny resulting from these two crosses and on WT males. All courtship tests took place 1–4 h after lights on. They were carried out under a dim red light (25W with a Kodak Safe-light filter n°1) to remove all visual stimuli [17] and with decapitated object flies to remove acoustic inhibitory signals [16]. Briefly, 5-day-old courting males were individually aspirated (without anesthesia) under a watch glass used as an observation chamber (1.6 cm^3^). After 5 min, a 5-day-old decapitated object fly was introduced and the courtship index (CI) directed toward each object fly was measured for a total duration of 10min. The CI is the proportion of time that the courting male spends actively courting (wing vibration, licking and attempting copulation) the object fly (no qualitative difference was noted between the courtship sequences of the different courting males). Each chemical to be tested was diluted in the appropriate solvent (pentane for 7-T ; 50% ethanol for caffeine ; 25% ethanol for quinine; water for berberine) at a concentration adjusted to standardize the volume of solvent deposited on each object fly (0.8 µl). For each substance, we tested several doses varying between either 250 ng (for 7-T and berberine) or 500 ng (for caffeine and quinine) and 2250 ng. These extreme doses correspond to the following molarity ranges (in mM): 2-18 for 7-T; 2.53-11.40 for caffeine; 1.53-6.90 for quinine; 0.5-4.5 for berberine. Tests comparing males of different genotypes were performed simultaneously, over several days.

Proboscis Extension Reflex (PER) tests were always carrried out during the afternoon. One-day-old WT flies were first placed in a vial with fresh medium for one day and were then starved for 20–22 h in a vial plugged with a piece of Kimwipe soaked with water, before being tested. Flies fixed as in [24] were stimulated two or three times with 0.1M sucrose to confirm the robustness of their behavior. The PER was always elicited immediately after stimulation. For bilateral stimulation one leg was first touched with 10^−8^M 7-T or with 0.1M caffeine mixed in mineral oil (or with mineral oil alone) and the other leg was then immediately touched with 0.1M sucrose. Contact lasted less than one second, and more than two minutes were left between stimulations. After bilateral stimulation, both legs were checked to have a normal response to sucrose (data not shown).

### Electrophysiology

The proboscis was exposed and maintained in place between a glass plate and a rod mounted on a micromanipulator and oriented under a stereomicroscope (Leica M10, ×250, Germany). Labellar taste sensilla were stimulated for 2 sec by a micropipette with a 20 µm tip diameter containing the stimulus. Recordings were obtained from an electrolytically-sharpened tungsten microelectrode inserted at the base of taste sensilla (Hiroi *et al.* unpublished) connected to a custom-built preamplifier (×10) and amplified using the second channel of the CyberAmp 320 amplifier (×100, 8 dB Bessel band-pass filter = 0.1 Hz-2800 Hz). The electrical signals were sampled at 10 kHz on a computer and analyzed using dbWave (http://quasimodo.versailles.inra.fr/deterrents/tk/dbwave/), to detect and sort spikes.

### Chemical Synthesis

The target compound was prepared as in [20]: coupling of the lithium salt of 1-octyne (Aldrich) with 1-bromopentadecane (Aldrich) produced 7-tricosyne which was transformed to *Z*-7-tricosene upon hydrogenation, using P-2 nickel as the catalyst. The crude product was purified by chromatography on silica showing a final purity of 99% (checked by a GC-MS ; Shimatzu QP2010).

## References

[1] Darwin C (1871). Descent of man, and selection in relation to sex..

[2] Hall JC (1994). The mating of a fly.. Science.

[3] Billeter JC, Rideout EJ, Dornan AJ, Goodwin SF (2006). Control of male sexual behavior in Drosophila by the sex determination pathway.. Curr Biol.

[4] Ferveur JF (2005). Cuticular hydrocarbons: their evolution and roles in Drosophila pheromonal communication.. Behav Genet.

[5] Howard RW, Blomquist GJ (2005). Ecological, behavioral and biochemical aspects of insect hydrocarbon.. Annu Rev Entomol.

[6] Ferveur JF, Sureau G (1996). Simultaneous influence on male courtship of stimulatory and inhibitory pheromones produced by live sex-mosaic *Drosophila melanogaster*.. Proc Biol Sci.

[7] Antony C, Jallon JM (1982). The chemical basis for sex recognition in *Drosophila melanogaster*.. J Insect Physiol.

[8] Jallon JM (1984). A few chemical words exchanged by Drosophila during courtship and mating.. Behav Genet.

[9] Stocker RF (1994). The organization of the chemosensory system in *Drosophila melanogaster*: a review.. Cell Tissue Res.

[10] Bray S, Amrein H (2003). A putative Drosophila pheromone receptor expressed in male-specific taste neurons is required for efficient courtship.. Neuron.

[11] Xu A, Park SK, D'Mello S, Kim E, Wang Q (2002). Novel genes expressed in subsets of chemosensory sensilla on the front legs of male Drosophila melanogaster.. Cell Tissue Res.

[12] Svetec N, Ferveur JF (2005). Social experience and pheromonal perception can change male-male interactions in Drosophila melanogaster.. J Exp Biol.

[13] Park SK, Mann KJ, Lin H, Starostina E, Kolski-Andreaco A (2006). A Drosophila protein specific to pheromone-sensing gustatory hairs delays males' copulation attempts.. Curr Biol.

[14] Hodgson ES, Lettvin JY, Roeder KD (1955). Physiology of a primary chemoreceptor unit.. Science.

[15] Ozaki M, Wada-Katsumata A, Fujikawa K, Iwasaki M, Yokohari F (2005). Ant nestmate and non-nestmate discrimination by a chemosensory sensillum.. Science.

[16] Ferveur JF, Stortkuhl KF, Stocker RF, Greenspan RJ (1995). Genetic feminization of brain structures and changed sexual orientation in male Drosophila.. Science.

[17] Boll W, Noll M (2002). The Drosophila *Pox neuro* gene: control of male courtship behavior and fertility as revealed by a complete dissection of all enhancers.. Development.

[18] Marcillac F, Bousquet F, Alabouvette J, Savarit F, Ferveur JF (2005). A mutation with major effects on *Drosophila melanogaster* sex pheromones.. Genetics.

[19] Savarit F, Sureau G, Cobb M, Ferveur JF (1999). Genetic elimination of known pheromones reveals the fundamental chemical bases of mating and isolation in Drosophila.. Proc Natl Acad Sci U S A.

[20] Schmitt U, Lübke G, Francke W (1991). Tarsal secretion marks food sources in bumblebees (Hymenoptera: Apidae).. Chemoecology.

[21] Shanbhag SR, Park SK, Pikielny CW, Steinbrecht RA (2001). Gustatory organs of *Drosophila melanogaster*: fine structure and expression of the putative odorant-binding protein PBPRP2.. Cell Tissue Res.

[22] Meunier N, Marion-Poll F, Rospars JP, Tanimura T (2003). Peripheral coding of bitter taste in Drosophila.. J Neurobiol.

[23] Hiroi M, Meunier N, Marion-Poll F, Tanimura T (2004). Two antagonistic gustatory receptor neurons responding to sweet-salty and bitter taste in Drosophila.. J Neurobiol.

[24] Kimura K, Shimozawa Y, Tanimura T (1986). Isolation of Drosophila mutant with abnormal proboscis extension reflex.. J Exp Zool.

[25] Thorne N, Chromey C, Bray S, Amrein H (2004). Taste perception and coding in Drosophila.. Curr Biol.

[26] Wang Z, Singhvi A, Kong P, Scott K (2004). Taste representations in the Drosophila brain.. Cell.

[27] Moon SJ, Kottgen M, Jiao Y, Xu H, Montell C (2006). A taste receptor required for the caffeine response in vivo.. Curr Biol.

[28] Zufall F, Leinders-Zufall T (2000). The cellular and molecular basis of odor adaptation.. Chem Senses.

[29] Rezaval C, Werbajh S, Fernanda Ceriani M (2007). Neuronal death in Drosophila triggered by GAL4 accumulation.. Eur J Neurosci.

[30] Lin DY, Zhang SZ, Block E, Katz LC (2005). Encoding social signals in the mouse main olfactory bulb.. Nature.

[31] Liberles SD, Buck LB (2006). A second class of chemosensory receptors in the olfactory epithelium.. Nature.

